# Morphological and Molecular Gonadal Sex Differentiation in the Wild Japanese eel *Anguilla japonica*

**DOI:** 10.3390/cells11091554

**Published:** 2022-05-05

**Authors:** Moemi Horiuchi, Seishi Hagihara, Manabu Kume, Daichi Chushi, Yuya Hasegawa, Hikaru Itakura, Yoh Yamashita, Shinji Adachi, Shigeho Ijiri

**Affiliations:** 1 Graduate School of Fisheries Sciences, Hokkaido University, Hakodate 041-8611, Hokkaido, Japan; horiuchim@irago.co.jp (M.H.); daichi_chushi@kyokuyo.co.jp (D.C.); y-hasegawa1031@frontier.hokudai.ac.jp (Y.H.); s-adachi@fish.hokudai.ac.jp (S.A.); 2 Atmosphere and Ocean Research Institute, The University of Tokyo, Kashiwa 277-8564, Chiba, Japan; s-hagihara@aori.u-tokyo.ac.jp (S.H.); hikaruitakura@aori.u-tokyo.ac.jp (H.I.); 3 Field Science Education and Research Center, Kyoto University, Kyoto 606-8502, Kyoto, Japan; kume.manabu.6x@kyoto-u.ac.jp (M.K.); yamashita.yoh.4c@kyoto-u.ac.jp (Y.Y.)

**Keywords:** *Anguilla japonica*, Japanese eel, gonadal sex differentiation, sex differentiation-related gene, aromatase

## Abstract

Most cultured Japanese eels (*Anguilla japonica*) show male sex differentiation; however, natural gonadal sex differentiation has not been evaluated. In this study, this process was characterized in wild eels. Differentiated ovaries and testes were observed after the eels grew to 320 and 300 mm in total length, respectively. The youngest ovary and testis appeared at 3 and 4 years old, respectively; however, undifferentiated gonads were found up to 7 years, suggesting that sex differentiation was triggered by growth rather than aging. *gsdf*, *amh*, *foxl2b* and *foxl3b* were highly expressed in the testes, whereas *figla*, *sox3*, *foxn5*, *zar1,* and *zp3* were highly expressed in the ovaries. The expression of *cyp19a1a* and *foxl2a* did not differ significantly between the testis and ovary. In the ovaries, the *cyp19a1a* and *foxl2a* levels were highest in the early stages, suggesting that their function is limited to early ovarian differentiation. The *foxn5*, *zar1* and *zp3* levels tended to increase in the later stages, suggesting that they function after the initiation of ovarian differentiation. In undifferentiated gonads, dimorphic gene expression was not observed, suggesting that the molecular sex differentiation phase is short and difficult to detect. These findings provide the first demonstration of the whole course of natural gonadal sex differentiation in eels at molecular and morphological levels.

## 1. Introduction

In vertebrates, sex differentiation is determined by genetic and/or environmental factors. Most mammals have the XX/XY sex-determination system, and birds have the ZZ/ZW system. In these animals, sex is determined by sex chromosomes, called genetic sex determination (GSD) [1,2]. In contrast, sex is determined by environmental conditions in reptiles and amphibians during their sex differentiation period, termed environmental sex determination (ESD), such as temperature-dependent sex determination [3,4].

Fish show both GSD and ESD [5,6,7]. In medaka (*Oryzias latipes*), several studies have identified the sex-determining gene *dmy/dmrt1Y*, which is the second known vertebrate sex-determining gene, after *sry* in humans [8,9]. In tiger pufferfish (*Takifugu rubripes*) and Nile tilapia (*Oreochromis niloticus*), sex is determined by single-nucleotide polymorphisms in *amhr2* and *amh*, respectively, located on the Y chromosome [10,11]. Recently, in yellowtail (*Seriola quinqueradiata*), a single-nucleotide polymorphism in a gene encoding a steroidogenic enzyme, *hsd17b1,* on the W chromosome was identified as the sex-determining gene. This study suggested that the enzymatic activity of *hsd17b1* encoded on W to produce estradiol-17β (E2) was higher than that on the Z chromosome, suggesting that it leads to E2 production in ZW fish to induce ovarian differentiation [12]. Japanese flounder (*Paralichthys olivaceus*) undergo GSD; however, their sex is strongly male-biased in high-temperature conditions during the gonadal differentiation period [13]. In medaka, high temperatures lead to stress-induced cortisol synthesis [14] and the expression of steroidogenic enzymes involved in androgen synthesis, resulting in the sex reversal of XX fish to males [15]. Thus, fish show substantial interspecific variation in sex determination systems.

Sex differentiation in eels in aquaculture conditions may be influenced by environmental factors, such as rearing density. Most European eels (*Anguilla anguilla*) in the glass eel stage develop into males in aquaculture farms [16]. Under experimental conditions, the ratio of males increases with an increasing rearing density [17]. Japanese eels also differentiate mostly to males in aquaculture conditions [18]. However, the mechanism underlying this male bias is unknown in cultured eels. Thus, under captive conditions, estrogen (E2) is fed to glass eels to produce females and for their sexual maturation and production of eggs that are eventually utilized for artificial maturation [19]. However, ovarian differentiation induced by exogenous E2 treatment is not identical to natural ovarian differentiation. Owing to the difficulty in obtaining naturally differentiating females under experimental conditions, investigating the entire course of gonadal differentiation in wild eels is necessary to understand this process.

In the wild, glass eels migrate from the sea to freshwater sources, such as rivers or lakes. They then grow into the yellow eel stage wherein gonadal differentiation occurs. A study on wild and captive European eels reported that gonadal sex differentiation occurs when their size reaches 220–300 mm [20]. In Japanese eels under experimental conditions, apparent testes and E2-induced differentiated ovaries were observed after they grew to 350 and 180 mm, respectively, within six months of rearing glass eels [21]. However, no studies have described the whole course of molecular and morphological gonadal sex differentiation in wild Japanese eel. Further, the timing of morphological sex differentiation has not been determined.

In almost all fish studied to date, estrogen production is the primary trigger for the initiation of ovarian differentiation [22,23]. In Nile tilapia, the aromatase level encoded by *cyp19a1a* is significantly higher in the undifferentiated gonads of genetic females (XX) than that in males (XY) 20 days before the start of morphological ovarian differentiation [24]. This female-specific *cyp19a1a* expression was regulated by a transcriptional factor, *foxl2*, which also shows female-specific expression [25]. In addition, both *foxl2*^−/−^ and *cyp19a1a*^−/−^ XX fish of Nile tilapia mutant lines showed a decrease in E2 levels leading to female-to-male sex reversal [26]. In XY undifferentiated gonads, the expression of these two genes was constantly very low or undetectable, resulting in failed estrogen production [24]. Estrogen production simultaneously suppressed testicular differentiation by inhibiting the *amh*, *gsdf* and *dmrt1* expression [27]. Similarly, in Japanese eels, the E2 treatment-induced *cyp19a1a* expression is higher in early differentiating ovaries than that in the testes [28]. In captive European eels that were not treated with E2, the *cyp19a1a* expression was higher in differentiating ovaries than that in differentiating testes [29]. Furthermore, a follow-up study demonstrated that the *foxn5*, *zar1* and *zp3* expression levels are higher in the differentiating ovary. In contrast, the *amh*, *dmrt1*, *gsdf* and *pre-miR202* levels are higher in the differentiating testis [30]. These results revealed that gene expression differs between apparently differentiated ovaries and testes; however, expression differences at the early gonadal differentiation stage remain unknown. In captive Japanese eel, the *dmrt1* and *sox9a* expression increased significantly during testicular differentiation; in contrast, the *figla* and *sox3* expression increased during ovarian differentiation in E2-treated eels [21]. Although E2 feeding induces ovarian differentiation, gene expression patterns in E2-induced ovaries may not reflect natural ovarian differentiation in eels. 

The entire course of molecular sex differentiation has not been investigated, especially during natural ovarian differentiation owing to the difficulty in obtaining sufficient gonadal samples from the wild. In this study, we characterized the timing of gonadal sex differentiation and the expression dynamics of sex differentiation-related genes in wild Japanese eels. 

## 2. Materials and Methods

### 2.1. Sample Collection

**Figure 1:**
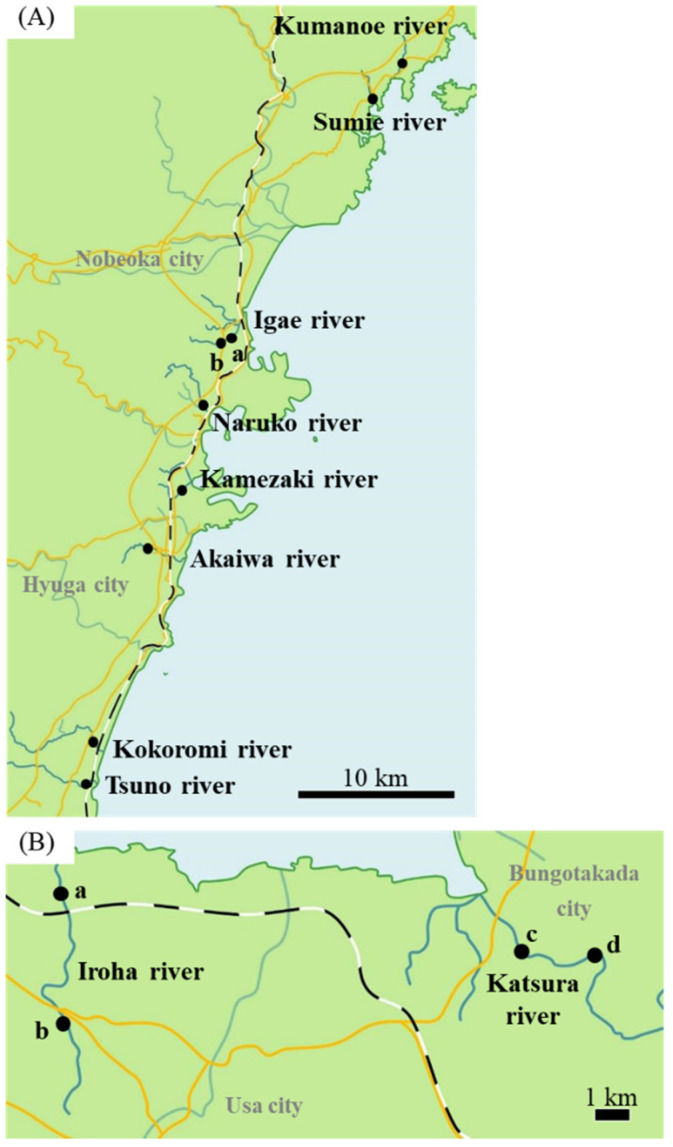
Sampling area. (**A**) Sampling area in Miyazaki River. In the Igae River, samples were collected from two locations (a, b). (**B**) Sampling area in Oita River. In Iroha and Katsura rivers, samples were collected from two locations (Iroha River: a, b. Katsura River: c, d.). Closed circles represent sampling areas. Scale bar: 10 km (**A**); 1 km (**B**).

In total, 189 wild Japanese eels (185–425 mm) were collected in Kumanoe (n = 23), Sumie (n = 13), Igae (n = 59), Naruko (n = 5), Kamezaki (n = 10), Akaigawa (n = 5), Kokoromi (n = 2), and Tsuno (n = 9) rivers in Miyazaki prefecture (Figure 1A) and Iroha (n = 33) and Katsura (n = 30) rivers in Oita prefecture between October 29 and November 7 in 2018 (Figure 1B). Eels were collected by electrofishing (LR-20B, SmithRoot, Vancouver, WA, USA). Then, 2-phenoxyethanol was applied as anesthesia, and eels were euthanized. Sampling was performed within 30 min to obtain accurate mRNA levels. Gonads from the right side and otoliths were isolated. Gonads were treated immediately with RNA later solution (Thermo Fisher Scientific Inc., Waltham, MA, USA) to stabilize and protect the RNA and to prevent the degradation caused by the stress responses after capture. A portion of the body with gonads on the left side was fixed in Bouin’s solution. Eel sampling was conducted in accordance with the guidelines of National University Corporation Hokkaido University Regulations on Animal Experimentation, Japan.

### 2.2. Histological Observation

The body samples were fixed with Bouin’s solution at room temperature overnight, after which fixatives were replaced with 70% ethanol. The samples were embedded in paraffin, cross-sectioned at 5–6 µm, and used for hematoxylin-eosin (HE) staining. The cross-sectional width of ovaries was measured using ImageJ software [31].

### 2.3. Age Determination by Otoliths

Otoliths were embedded using polyester resin and cross-sectioned by MARINO RESEARCH Co., Ltd. (Kuwana, Mie, Japan). Then, etching was performed with 1% hydrochloric acid, followed by staining with toluidine blue overnight. The age was determined by counting the number of annual rings. The relationship between TL and percentage of morphologically sex-differentiated eels was described using a logistic equation.

### 2.4. RNA Extraction and cDNA Synthesis

Total RNA was extracted using the ISOGEN reagent (Nippon Gene Co., Ltd., Toyama, Japan). Then, 650 ng of total RNA from each gonad was reverse transcribed in a final volume of 20 μL using 48 ng of random primers (Invitrogen, Carlsbad, CA, USA) and the reverse transcriptase ReverTra Ace (200 U) (Toyobo, Osaka, Japan).

### 2.5. Quantitative PCR Analysis

Quantitative PCR (qPCR) was performed with PowerUp SYBR Green Master Mix (Applied Biosystems, Waltham, MA, USA) and the StepOnePlus Real-Time PCR System (Thermo Fisher Scientific Inc.). Reactions were performed in 10 µL, including 2 µL of a 30-fold dilution of cDNA. mRNA levels of sex differentiation-related genes (*cyp19a1a*, *foxl2a*, *figla*, *sox3*, *foxn5*, *zar1*, *zp3*, *gsdf*, *amh*, *foxl2b* and *foxl3b*) were measured using primers listed in Table 1. Two housekeeping genes (*ef1a*, *β-actin*) were measured in samples from ovaries and testes collected during sex differentiation. Both genes showed stable expression (data not shown), and *β-actin* was used as the reference gene in this study. mRNA levels were normalized using the calibration-curve method.

### 2.6. Statistical Analyses

Statistical analyses were carried out using Excel Ver 7.0. Relationships between gonadal sex differentiation, body size, and age were investigated using Tukey–Kramer tests, and differences were considered significant at *P* < 0.05. mRNA levels of sex differentiation-related genes calculated by the calibration-curve method were compared between the ovary and testis with the Mann–Whitney U test, and differences were considered significant at *P* < 0.01.

## 3. Results

### 3.1. Gonadal Sex Differentiation of Wild Japanese eel

**Figure 2:**
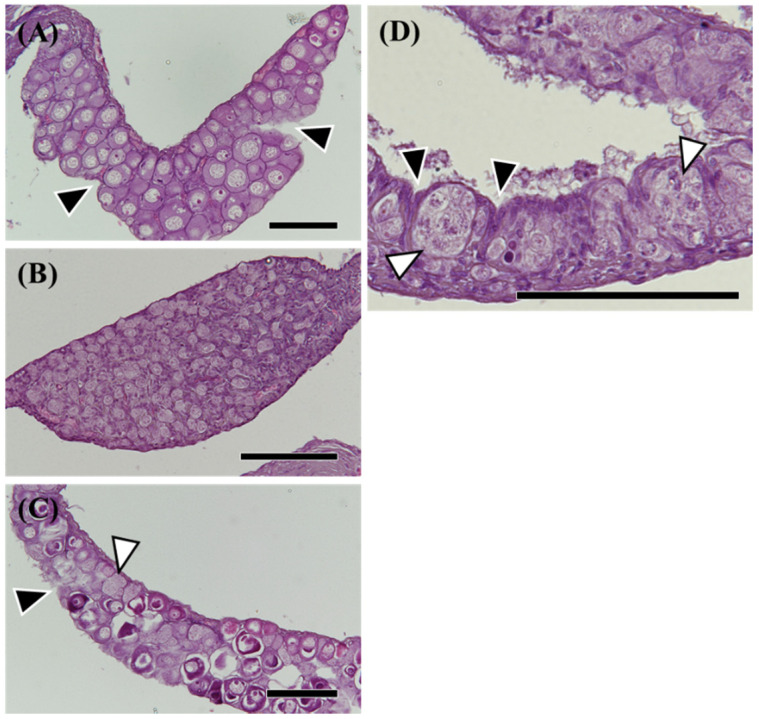
Hematoxylin-eosin (HE) staining of morphological sex-differentiated gonads in the wild Japanese eel *Anguilla japonica*. (**A**,**C**) Ovary. (**B**) Testis. (**D**) Differentiating ovary. Total lengths are (**A**) 342, (**B**) 306, (**C**) 257, (**D**) 297 mm. Arrowheads, notches in the epithelium of the ovary. White arrowheads, active germ cell growth. Scale bar, 100 µm.

**Figure 3:**
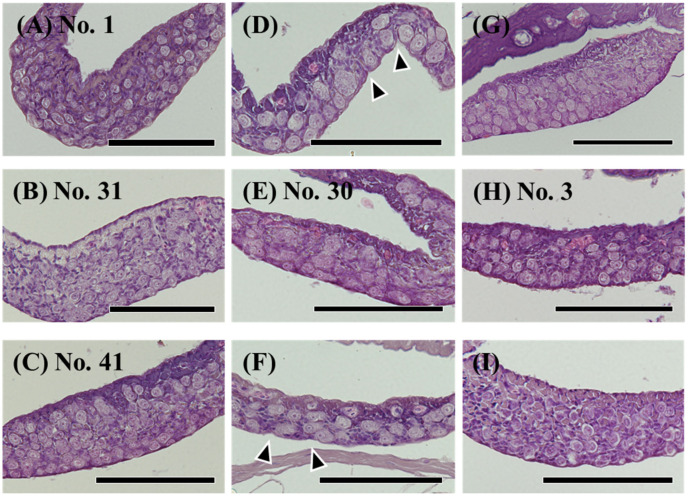
Hematoxylin-eosin (HE) staining of undifferentiated gonads in the wild Japanese eel *Anguilla japonica*. (**A**–**C**) Gonads of testis-like structures. (**D**–**F**) Gonads of ovary-like structures. (**G**–**I**) Undifferentiated gonads. Total lengths are (**A**) 250, (**B**) 305, (**C**) 313, (**D**) 281, (**E**) 299, (**F**) 310, (**G**) 251, (**H**) 257, (**I**) 318 mm. Arrowheads, notches in the epithelium of the ovary. Individual numbers (Nos.) correspond to those in Table 5 and Figures 10 and 11. Scale bar, 100 µm.

**Figure 4:**
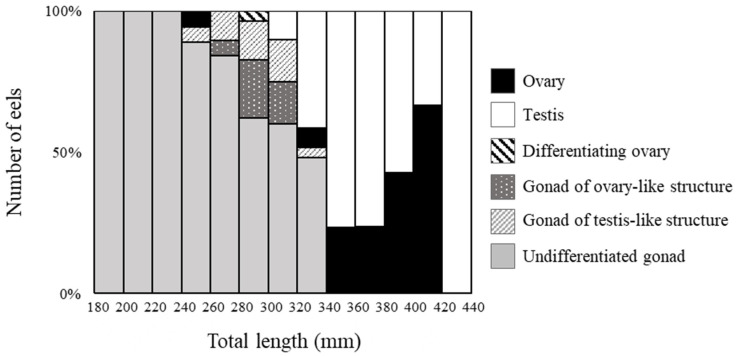
Percentage of morphological sex-differentiated eels with respect to body growth. Gonadal development was classified into six stages, and eels of each size were counted.

Among 189 eels (TL 185–425 mm), ovaries were observed in 19 eels (Figure 2A), and testes were observed in 59 eels (Figure 2B). Most differentiated gonads (with an apparent ovary or testis) were observed in eels over 320 mm in TL, except for a 257 mm eel with a differentiated ovary (Figure 2C) and a 297 mm eel with an early-differentiating ovary (Figure 2D). Additionally, 110 eels had undifferentiated gonads (Figure 3); however, gonads containing many germ cells were identified as a testis-like structure (Figure 3A–C). In addition, slight notches in the epithelium were identified as ovary-like structures (Figure 3D–F). Among 56 eels between 250 and 300 mm in length, testis-like and ovary-like structures were observed in seven eels each. Similarly, among 68 eels between 300 and 350 mm, testis-like and ovary-like structures were observed in four and three eels, respectively (Figure 4). In this study of wild Japanese eels, intersexual gonads were not observed.

### 3.2. Sex Ratio

In all rivers, males (eels with testis) were observed more frequently than females (eels with ovaries). Our survey from 10 rivers suggested that the sex ratio did not vary among locations (Table 2). The average TL and age were 352.4 ± 2.2 mm and 5.5 ± 1.2 years, respectively, for males and 358.0 ± 3.2 mm and 5.3 ± 1.6 years, respectively, for females. The differences in these parameters between males and females were not statistically significant.

### 3.3. Relationships between Gonadal Sex Differentiation, Body Size and Age

**Figure 5:**
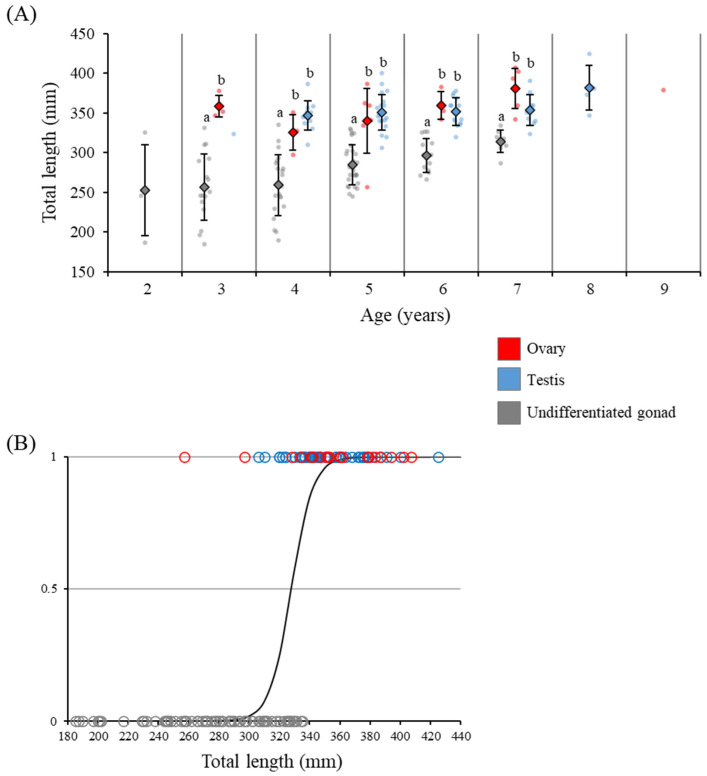
Relationship between the stage of gonadal development, TL, and age in the wild Japanese eel *Anguilla japonica*. (**A**) Different letters indicate significant differences (*P* < 0.05) relative to the ovary, testis, and undifferentiated gonad. (**B**) TL with respect to morphological sex differentiation. Y, probability of gonadal sex differentiation.

The TL of individuals with undifferentiated gonads, ovaries, and testes were plotted against age; even among eels of the same age, the TL of eels after morphological sex differentiation was significantly larger than that in the undifferentiated state (Figure 5A). In addition, comparing gonadal developmental stages and the TL using logistic regression, the TL corresponding to 10% morphological sex differentiation was 312.4 mm, and the TL for 90% was 343.5 mm (Figure 5B).

### 3.4. Expression of Sex Differentiation-Related Genes in Relation with Gonadal Sex Differentiation

**Figure 6:**
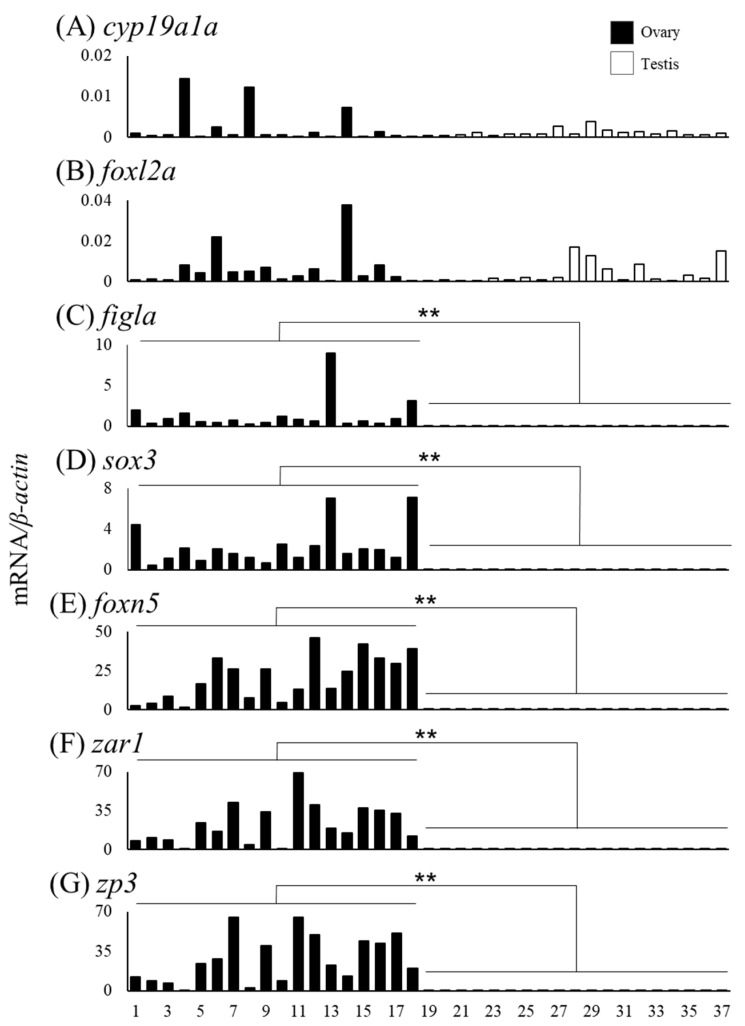
Messenger RNA levels of sex differentiation-related genes (*cyp19a1a*, *foxl2a*, *figla*, *sox3*, *foxn5*, *zar1* and *zp3*) in the ovaries and testes of the wild Japanese eel *Anguilla japonica.* (**A**) *cyp19a1a*, (**B**) *foxl2a*, (**C**) *figla*, (**D**) *sox3*, (**E**) *foxn5*, (**F**) *zar1* and (**G**) *zp3* mRNA levels in the ovary and testis. Each column indicates mRNA levels normalized against *β-actin* expression. TL in the ovary and testis (Nos. 1–37); for details, refer to Table 3. ** *P* < 0.01.

**Figure 7:**
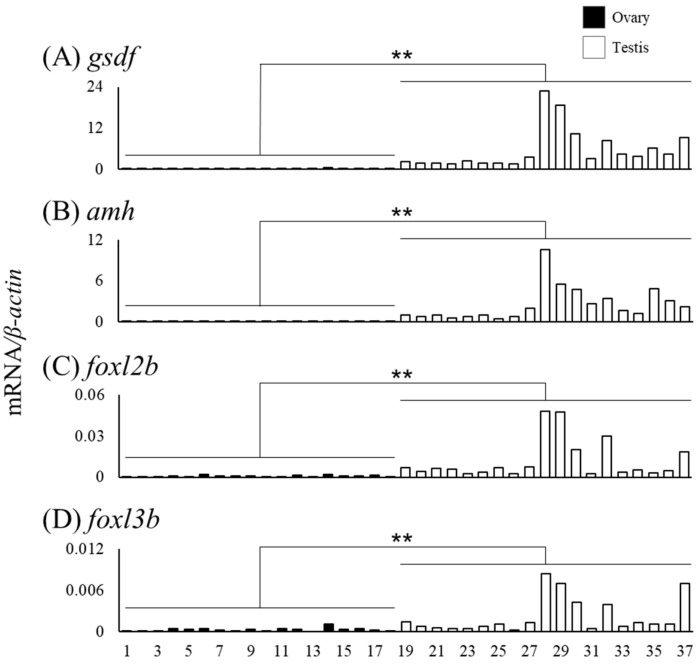
Messenger RNA levels of sex differentiation-related genes (*gsdf*, *amh*, *foxl2b* and *foxl3b*) in the ovary and testis of the wild Japanese eel *Anguilla japonica.* (**A**) *gsdf*, (**B**) *amh*, (**C**) *foxl2b* and (**D**) *foxl3b* mRNA levels in the ovary and testis. Each column indicates mRNA levels normalized against *β-actin* expression. TL in the ovary and testis (Nos. 1–37); for details, see Table 3. ** *P* < 0.01.

We measured mRNA levels of sex differentiation-related genes (*cyp19a1a*, *foxl2a*, *figla*, *sox3*, *foxn5*, *zar1*, *zp3*, *gsdf*, *amh*, *foxl2b* and *foxl3b*) in the gonads of female (n = 18) and male eels (n = 19) (Table 3). The *cyp19a1a* mRNA levels were higher in ovaries of Nos. 4, 8, and 14 compared to that in the other eels, and the *foxl2a* levels were elevated in ovaries of Nos. 6 and 14 (Figure 6A,B). The *figla* and *sox3* mRNA levels showed substantial variation but were significantly higher in the ovary than in the testis (Figure 6C,D). The mRNA levels of *foxn5*, *zar1* and *zp3* also showed variation and were significantly higher in the ovary than in the testis. In contrast, the mRNA levels of *gsdf*, *amh*, *foxl2b* and *foxl3b* were significantly higher in the testis than in the ovary; in particular, eels exceeding the TL of No. 28 (337 mm) tended to express higher mRNA levels than those of smaller eels (Figure 7).

### 3.5. Relationship between the Expression of Sex Differentiation-Related Genes and Ovarian Developmental Stage

**Figure 8:**
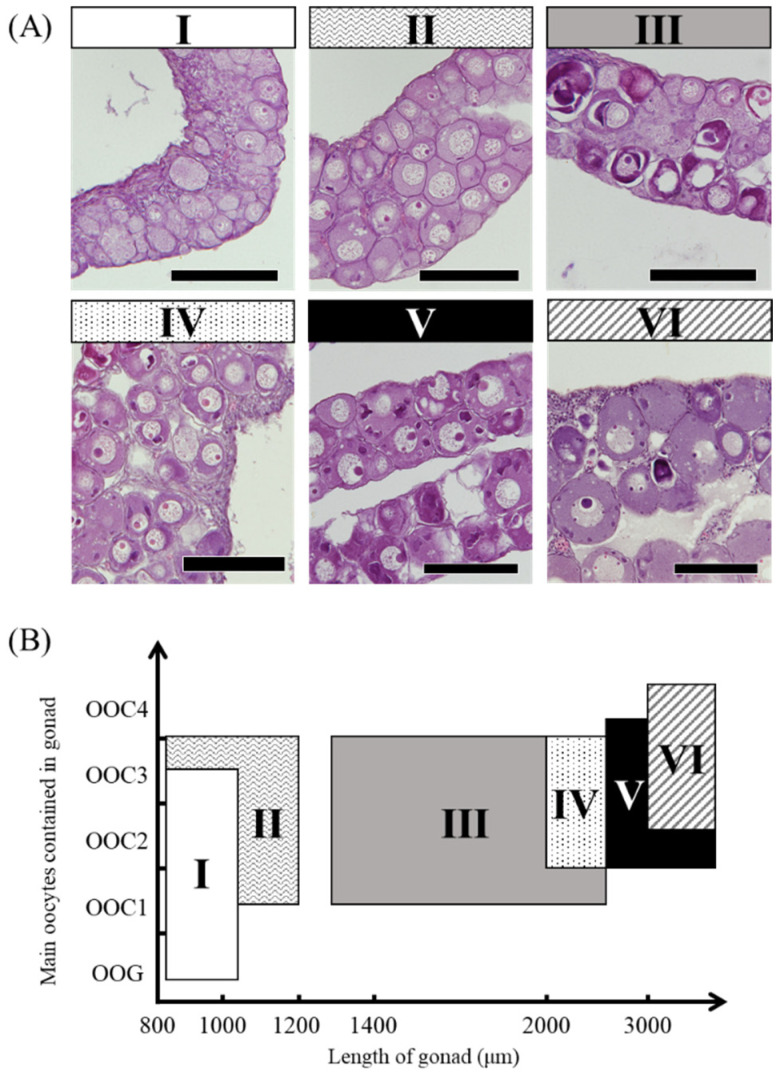
Ovarian developmental stages in the wild Japanese eel *Anguilla japonica.* (**A**) OOG: oogonial cyst, OOC1: cyst of early meiotic oocytes, OOC2: postpachytene oocyte, OOC3: early previtellogenic oocyte, OOC4: previtellogenic oocyte. Scale bar, 100 µm. (**B**) Distribution of groups Ⅰ–Ⅵ using ovarian developmental stages and the width of gonadal cross-sections.

**Figure 9:**
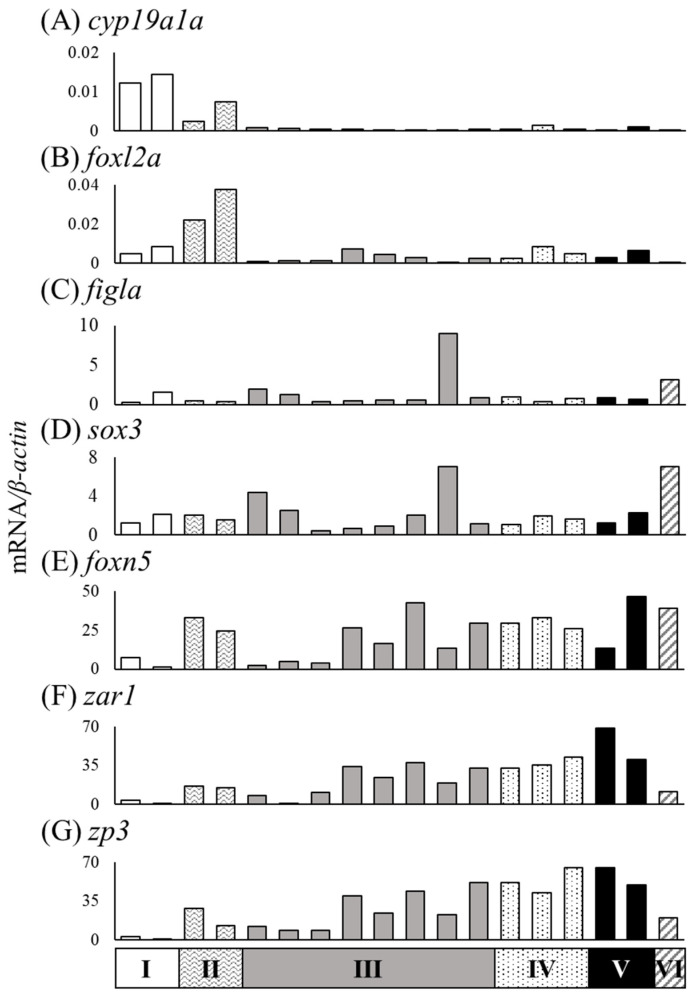
Relationships between stages of ovarian development and mRNA levels in gonads of the wild Japanese eel *Anguilla japonica.* (**A**) *cyp19a1a*, (**B**) *foxl2a*, (**C**) *figla*, (**D**) *sox3*, (**E**) *foxn5*, (**F**) *zar1* and (**G**) *zp3* mRNA levels in the ovaries of groups Ⅰ–Ⅵ. Each column indicates mRNA levels that are normalized against levels of *β-actin*. TL in eels of groups A–F; for details, refer to Table 4.

The mRNA expression of the sex differentiation-related genes *cyp19a1a*, *foxl2a*, *figla*, *sox3*, *foxn5*, *zar1* and *zp3* showed wider variation among 18 female eels than among male eels. We classified eels based on the ovarian development stage and the gonadal cross-sectional width (Ⅰ–Ⅵ) (Figure 8). The ovarian developmental stage of 18 ovaries classified as Ⅰ–Ⅵ was further evaluated by the distribution of the oogonial cyst (OOG), cyst of early meiotic oocytes (OOC1), postpachytene oocyte (OOC2), early previtellogenic oocyte (OOC3), and previtellogenic oocyte (OOC4) based on the study by Colombo and Grandi [20]. More than half of the gonads showed OOG and OOC1 cysts in group Ⅰ. In group Ⅱ, more than half of the gonads were an OOC3, and OOG cysts were no longer observed. Group Ⅲ showed the same developmental stage as Ⅱ; however, the cross-sectional width exceeded 1300 μm. In group Ⅳ, most gonads were an OOC3, and OOC1 was not observed. The developmental stage of group Ⅴ was similar to that of group Ⅳ; however, an OOC4 was observed. Group Ⅵ had a large amount of OOC4, and the cross-sectional width of gonads was over 3000 μm (Figure 8). The mRNA levels in 18 female eels were evaluated in groups Ⅰ–Ⅵ (Table 4). The mRNA levels of *cyp19a1a* tended to be high in group Ⅰ, and *foxl2a* tended to be high in group Ⅱ (Figure 9A,B). The *figla* and *sox3* mRNA levels showed variation within groups (Figure 9C,D). The mRNA levels of *foxn5*, *zar1* and *zp3* tended to be low in group Ⅰ and high in groups Ⅱ–Ⅵ (Figure 9E–G).

### 3.6. Expression of Sex Differentiation-Related Genes in Undifferentiated Gonads

**Figure 10:**
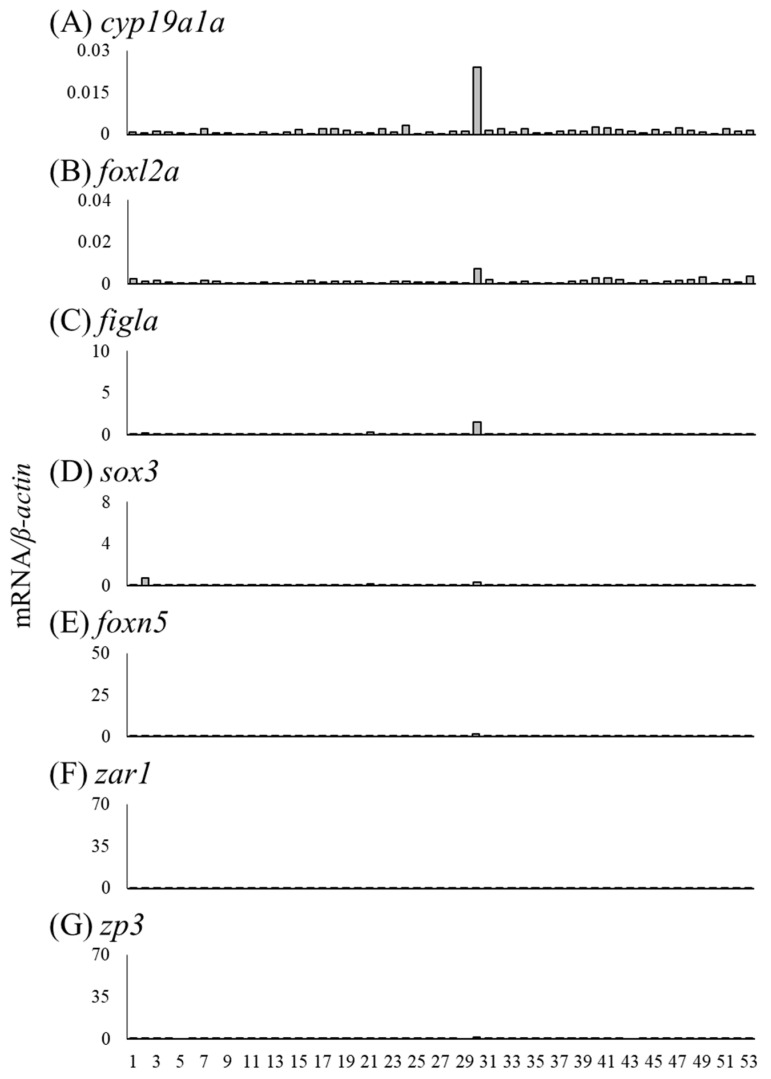
Messenger RNA levels of sex differentiation-related genes (*cyp19a1a*, *foxl2a*, *figla*, *sox3*, *foxn5*, *zar1* and *zp3*) in undifferentiated gonads of the wild Japanese eel *Anguilla japonica.* (**A**) *cyp19a1a*, (**B**) *foxl2a*, (**C**) *figla*, (**D**) *sox3*, (**E**) *foxn5*, (**F**) *zar1* and (**G**) *zp3* mRNA levels in undifferentiated gonads. Each column indicates mRNA levels normalized against levels of *β-actin*. TL in undifferentiated gonads, including testis-like and ovary-like structures (No. 1–53); for details, refer to Table 5.

**Figure 11:**
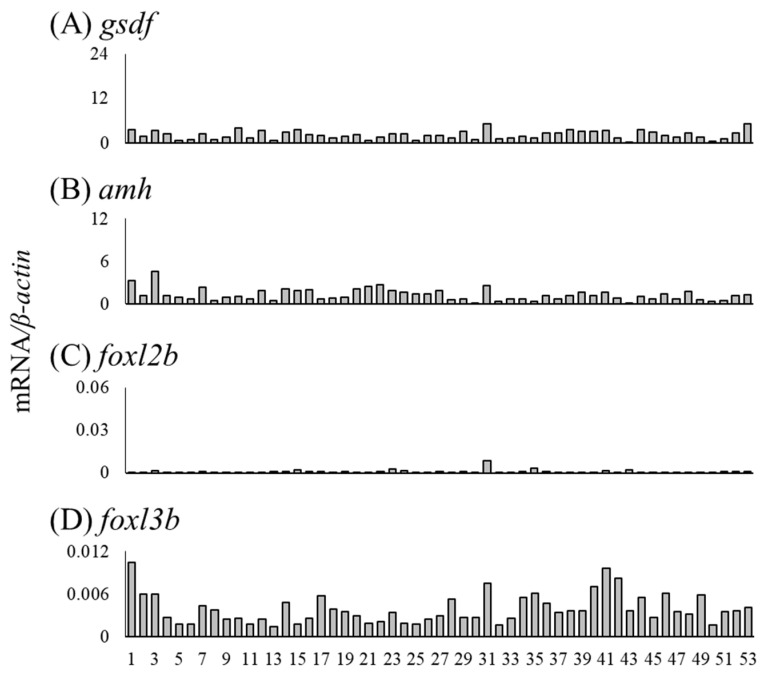
Messenger RNA levels of sex differentiation-related genes (*gsdf*, *amh*, *foxl2b* and *foxl3b*) in undifferentiated gonads of the wild Japanese eel *Anguilla japonica.* (**A**) *gsdf*, (**B**) *amh*, (**C**) *foxl2b* and (**D**) *foxl3b* mRNA levels in undifferentiated gonads. Each column indicates mRNA levels normalized against levels of *β-actin*. TL in undifferentiated gonads, including testis-like and ovary-like structures (Nos. 1–53); for details, see Table 5.

We investigated the mRNA levels of sex differentiation-related genes in undifferentiated gonads, including testis-like and ovary-like structures (Table 5). The mRNA levels of *cyp19a1a*, *foxl2a* and *figla* were higher in the gonads of No. 30 than in other undifferentiated gonads (Figure 10A–C). No. 30 was a gonad with an ovary-like structure (Table 5, Figure 3E). The mRNA levels of *sox3*, *foxn5*, *zar1* and *zp3* were low in all undifferentiated gonads (Figure 10D–G). In the gonad of No. 31, the mRNA levels of four genes (*gsdf*, *amh*, *foxl2b* and *foxl3b*) tended to be high (Figure 11). In addition, the mRNA levels of three genes (other than *foxl2b*) tended to be high in the gonad of No. 1 (Figure 11); in No. 41, only the mRNA level of *foxl3b* was high, as in No. 1 (Figure 11D). Nos. 1, 31 and 41 had gonads with a testis-like structure (Table 5, Figure 3A–C). Furthermore, among the individuals with undifferentiated gonads in this study, the mRNA levels of *amh* and *foxl3b* were high in the gonad of No. 3 (Figure 3H and Figure 11B,D). However, the mRNA levels of sex differentiation-related genes in these 53 eels with undifferentiated gonads were significantly lower than those in the female and male eels.

## 4. Discussion

Recent studies have rapidly improved our understanding of the molecular mechanisms underlying gonadal sex differentiation in teleost fish, such as Nile tilapia and medaka. However, studies on less-derived fish are lacking. Despite its commercial importance, gonadal sex differentiation in the genus *Anguilla* has not been characterized because most eels differentiate to males under captive conditions, and capturing wild eels is highly restricted. In this study, we collected 189 wild eels at various developmental stages from various rivers and investigated the whole process of morphological and molecular gonadal sex differentiation in detail, providing the first comprehensive analysis of natural sex differentiation in the Japanese eel.

Our histological observations revealed undifferentiated gonads, ovary-like structures (the beginning stage of ovarian differentiation), testis-like structures (the beginning stage of testicular differentiation), early differentiating ovaries (gonads, including clusters of active mitotic germ cell division), and differentiated ovaries and testes. Testis-like and ovary-like structures were found after a TL of 240 and 260 mm, respectively. Testis-like and ovary-like structures were not differentiated gonads; however, this morphological feature probably represents the beginning of testicular or ovarian differentiation. These structures were detected when the TL was between 240 and 320 mm, suggesting that gonadal sex differentiation is initiated within this range. Except for one eel with an ovary at a TL of 257 mm, ovaries and testes were observed after the eels reached a TL of 320 and 300 mm, respectively. After a TL of 340 mm, all eels had differentiated ovaries or testes, suggesting that morphological gonadal sex differentiation is completed by this size. Under experimental conditions, differentiated testes were observed after 350 mm, similar to observations in wild eels; however, differentiated ovaries were observed after 180 mm, which is much smaller than the corresponding TL in wild eels [21], suggesting that E2 treatment facilitates ovarian differentiation. Ovaries and testes appeared after 220–300 mm in European eels [20] and 250–270 mm in American eels (*Anguilla rostrata*) [32], suggesting that morphological gonadal sex differentiation occurs later in Japanese eels than in these two species.

In European eels, Colombo and Grandi [20] suggested that the undifferentiated gonad may differentiate into the ovary directly or through the Syrski organ (intersexual organ), which includes oocytes in a testis-like structure. Later, Geffroy [29] suggested that the ovary differentiates directly from undifferentiated gonads, and the testis differentiates directly from undifferentiated gonads and/or intersexual organs containing degenerating oocytes. However, in wild Japanese eels, in the present study and our previous study of 100 wild eels with sizes of 300–400 mm (unpublished data) did not reveal any cases of intersexual organs. Therefore, unlike wild European eels, wild Japanese eels may show direct ovary and testis differentiation from undifferentiated gonads without an intersexual stage. In the captive Japanese eel, simultaneous rearing of multiple eels increases the serum cortisol level [33], and the rearing in high density induces a stress response [34]. In addition, under experimental conditions, Jeng [21] observed a number of intersexual organs. It has been proposed that the Japanese eel becomes male due to stress, and intersexual organs may emerge due to the sex reversal of a genetic female to a phenotypic male.

Other than growth, aging may be related to other factors that trigger the initiation of gonadal sex differentiation; this has not been investigated in wild eels. In this study, ovaries were observed in individuals as young as 3 years old, and testes were observed at 4 years old. 

At 3 and 4 years, undifferentiated eels were significantly smaller than sexually differentiated eels. Undifferentiated eels were seen as late as 7 years, at an average length of 310 mm, which was significantly smaller than the length of differentiated eels at this age. Under aquaculture conditions, Japanese eels show testis differentiation within 1 year [21,28], probably due to faster growth by saturation feeding. These observations suggest that aging is not the main trigger for gonadal sex differentiation, especially when observing undifferentiated eels as old as 7 years. An exceptionally small eel at 257 mm TL with an ovary containing many oocytes was 5 years old, suggesting that old age could not explain the induction of ovarian differentiation in the small eel.

Differentiating testes and ovaries express genes, termed sex differentiation-related genes, responsible for developing and maintaining each gonad. In the testis, *gsdf*, *amh*, *foxl2b* and *foxl3b* expression levels were significantly higher than those in the ovary. In Japanese eels in aquaculture conditions, the *amh* and *gsdf* expression was much higher in the testis than in the E2-induced ovary at an early developmental stage [28]. In European eels in aquaculture conditions, the expression of these two genes was much higher in the testis than in the naturally differentiated ovary at an early developmental stage [30]. In Nile tilapia, the *amh* and *gsdf* levels were higher in undifferentiated XY gonads compared to those in XX undifferentiated gonads. Either of the gene-disrupted XY tilapia differentiates ovaries, suggesting that these genes are testis inducing [11,35]. Consistent with these findings, in eels, *amh* and *gsdf* show high expression in early testes under both captive and wild conditions, suggesting that these two genes are essential for testis differentiation and development in eels. Wu [36] reported that *foxl3b* was expressed in Japanese eel testicular germ cells, and expression levels were higher in the control group than in the E2-treated group. Our results demonstrated that not only *foxl3b* but also *foxl2b* expression is higher in the developing testis than in the ovary in wild eels. This is the first observation of elevated *foxl2b* expression in differentiating testes in fish. The functions of these genes are unclear; however, the expression profile suggested that they have vital roles in eel testis differentiation and development.

Three and one developing ovaries showed elevated *cyp19a1a* and *foxl2a* expression levels, respectively; however, overall significant differences were not detected between the ovary and testis. In aquaculture eels, the *cyp19a1a* and *foxl2a* expression levels are significantly higher in the testis than in the E2-induced ovary [28]. The results of the present study suggested that high *cyp19a1a* and *foxl2a* expression are characteristic features of the E2-treated ovary and are not found in the naturally developing ovary. The *figla*, *sox3*, *foxn5*, *zar1* and *zp3* genes showed significantly higher expression in the ovary than in the testis. We further examined gene expression patterns during the early development of the ovary classified by fine histological observations from stages Ⅰ to Ⅵ. The *cyp19a1a* and *foxl2a* levels tended to be high at early stages of Ⅰ and Ⅱ, respectively, and low at later developmental stages. In European eels raised under aquaculture, the *cyp19a1a* expression tended to be higher in the early stage of ovarian development, corresponding to stage Ⅱ, than in more highly developed ovaries, corresponding to stage Ⅵ; however, these levels were not significantly higher than those in undifferentiated gonads [29]. Collectively, our results suggest that *cyp19a1a* and *foxl2a* expression is slightly elevated during the early ovarian developmental stage and is then low until the beginning stage of cortical alveoli. In contrast, the expression levels of *foxn5*, *zar1* and *zp3* tended to be elevated at later developmental stages. The *foxn5* gene (*forkhead box N5*) is involved in the embryonic development in mice [37], and higher expression in the ovary has been reported in marine medaka (*Oryzias melastigma*) [38]. Zar functions as an RNA-binding protein and plays a role in regulating maternal mRNA translation in African clawed frogs (*Xenopus laevis*) [39]. In zebrafish (*Danio rerio*), *zar1* (*zygotic arrest 1*) is required for very early oogenesis [40]. The specific expression of *zp3* (*zona pellucida glycoprotein 3*) in early oocytes has been reported in zebrafish [41]. Three genes seemed to be involved in later stages rather than early ovarian development in eels.

Before observable morphological gonadal sex differentiation, sex differentiation-related genes did not show dimorphic expression patterns in undifferentiated gonads, including testis-like and ovary-like structures. In Nile tilapia, the expression patterns of *cyp19a1a*, *foxl2* and *gsdf* showed evident sexual dimorphism after 5 days after hatching, 20 days before morphological gonadal differentiation begins [24]. In the undifferentiated gonads of Russian sturgeon, these three genes show distinct dimorphic expression patterns, and gonads with high *cyp19a1* and *foxl2* levels are presumptive female gonads, and those expressing high levels of *gsdf* are presumptive male gonads [42]. The sexual dimorphic gene expression period in Japanese eels may be short and challenging to detect. Further, 1 of the 53 eels (i.e., No. 30) showed very high *cyp19a1a* expression and moderately high *foxl2* and *figla* expression levels. This gonad showed an ovary-like structure and was a presumptive ovary just before differentiation; however, further investigations are required to clarify molecular sex differentiation in naturally differentiating eel gonads.

In this study, expression patterns of sex differentiation-related genes over the whole course of testicular and ovarian differentiation and early development in the wild Japanese eel were demonstrated for the first time. We revealed differences in expression patterns from those of E2-treated eels. We speculate that this study did not capture characteristic genes showing sexual dimorphic expression and triggering gonadal sex differentiation. Comparative analyses of global RNA expression profiles at the earliest stage of ovarian and testis development are required to identify these factors.

## Figures and Tables

**Table 1:** Primer sequences of sex differentiation-related genes used to qPCR.

Name	Primer sequence (5′-3′)
*cyp19a1a*_F	CAGAGAAGTTGGATGATGCTGACT
*cyp19a1a*_R	GCTCCCCGTGGTTCTGAGC
*foxl2a*_F	CCACCCACTCCTATGCCCTAT
*foxl2a*_R	GCCGACAGTCCTTTGACGTT
*figla*_F	ATGTTTTCCCGCCTGAAACG
*figla*_R	TCCAGCATACTGCCAGTCG
*sox3*_F	CTGGAACGAACGCTGTCAAC
*sox3*_R	GCTGGGATGCTGAGGGTATC
*foxn5*_F	CCTCGTCCAGCGAATATCTTC
*foxn5*_R	TGAGCGAGATTCAGCTTCC
*zar1*_F	CATCTCTGGAACCAATAAGGTG
*zar1*_R	CCACCCTGTACGGATTGAAC
*zp3*_F	GAGTTGGTGGTGGTCAAAG
*zp3*_R	CATACTGTCCACCATACAGCC
*gsdf*_F	ACAGTACAGGCTTGCAGTGCTG
*gsdf*_R	AGTTCTCCCAACCAAGATCGTT
*amh*_F	TCTCCTAGCAACGGATTGGC
*amh*_R	CACAGGAAGTGGAAAGTCCGG
*foxl2b*_F	CATTCTGACGCTCACCACCTT
*foxl2b*_R	CTTGTTGCGTCTGGAGAGGAA
*foxl3b*_F	CCGCTAAGTGGATTTCCGAAT
*foxl3b*_R	CCTGGATGGTGGAGGTGATT

**Table 2:** The number of eels sampled in each river, and gonadal development stage.

Sampling	Undifferentiated	Ovary-Like	Testis-Like	Ovarian	Ovary	Testis
Location		Structure	Structure	Differentiated		
Kumano River	11	1	4	-	1	6
Sumie River	9	-	-	-	-	4
Igae River_a	12	3	1	1	6	20
Igae River_b	7	2	-	-	2	5
Naruko River	4	-	-	-	1	-
Kamezaki River	8	-	-	-	-	2
Akaiwa River	1	-	1	-	-	3
Kokoromi River	1	-	-	-	1	-
Tsuno River	2	2	-	-	2	3
Iroha River_a	18	2	3	-	3	6
Iroha River_b	-	-	-	-	1	-
Katsura River_c	14	-	1	-	2	10
Katsura River_d	2	-	1	-	-	-

**Table 3:** Gonad samples of male and female Japanese eel used to qPCR.

No.	TL (mm)	Age (Years)	Male/Female	No.	TL (mm)	Age (Years)	Male/Female
1	257	5	Female	19	306	5	Male
2	328	4	Female	20	310	4	Male
3	334	5	Female	21	320	5	Male
4	341	5	Female	22	320	6	Male
5	342	7	Female	23	322	5	Male
6	342	6	Female	24	330	4	Male
7	347	3	Female	25	333	5	Male
8	351	4	Female	26	334	6	Male
9	353	6	Female	27	335	6	Male
10	360	7	Female	28	337	6	Male
11	360	5	Female	29	337	7	Male
12	362	5	Female	30	340	7	Male
13	378	3	Female	31	341	5	Male
14	379	9	Female	32	347	8	Male
15	383	6	Female	33	351	7	Male
16	387	5	Female	34	360	5	Male
17	394	7	Female	35	375	6	Male
18	407	7	Female	36	387	4	Male
				37	425	8	Male

**Table 4:** Classification of ovarian developmental stages in 18 female eels.

Group	TL (mm)	BW (g)	Age (Years)
**I**	351	37	4
	341	42	5
**II**	342	41	7
	379	49	9
**III**	257	13	5
	360	38	5
	328	25	4
	353	38	6
	342	36	6
	383	50	6
	378	37	3
	394	69	7
**IV**	334	28	5
	387	57	5
	347	36	3
**V**	360	56	7
	362	42	5
**VI**	407	64	7

**Table 5:** Undifferentiated gonad samples of Japanese eel used to qPCR.

No.	TL (mm)	Category	No.	TL (mm)	Category
1	250	Testis-like structure	27	297	Undifferentiated
2	251	Undifferentiated	28	297	Ovary-like structure
3	257	Undifferentiated	29	298	Ovary-like structure
4	258	Undifferentiated	30	299	Ovary-like structure
5	258	Undifferentiated	31	305	Testis-like structure
6	264	Testis-like structure	32	307	Undifferentiated
7	266	Undifferentiated	33	309	Ovary-like structure
8	266	Undifferentiated	34	310	Ovary-like structure
9	269	Undifferentiated	35	310	Undifferentiated
10	271	Testis-like structure	36	310	Undifferentiated
11	271	Undifferentiated	37	311	Undifferentiated
12	275	Undifferentiated	38	311	Ovary-like structure
13	275	Ovary-like structure	39	312	Undifferentiated
14	277	Undifferentiated	40	313	Testis-like structure
15	280	Undifferentiated	41	313	Testis-like structure
16	280	Undifferentiated	42	318	Undifferentiated
17	281	Ovary-like structure	43	320	Testis-like structure
18	287	Undifferentiated	44	320	Undifferentiated
19	287	Ovary-like structure	45	325	Undifferentiated
20	287	Undifferentiated	46	327	Undifferentiated
21	287	Testis-like structure	47	327	Undifferentiated
22	288	Testis-like structure	48	327	Undifferentiated
23	290	Testis-like structure	49	329	Undifferentiated
24	290	Undifferentiated	50	330	Undifferentiated
25	292	Testis-like structure	51	331	Undifferentiated
26	295	Ovary-like structure	52	334	Undifferentiated
			53	335	Undifferentiated

## Data Availability

All data are available within this article.
